# Hypothesis, analysis and synthesis, it's all Greek to me

**DOI:** 10.7554/eLife.43514

**Published:** 2019-02-20

**Authors:** Ioannis Iliopoulos, Sophia Ananiadou, Antoine Danchin, John PA Ioannidis, Peter D Katsikis, Christos A Ouzounis, Vasilis J Promponas

**Affiliations:** 1 Division of Basic Sciences, School of MedicineUniversity of CreteHeraklionGreece; 2 School of Computer ScienceUniversity of ManchesterManchesterUnited Kingdom; 3 Institute of Cardiometabolism and NutritionHôpital de la Pitié-SalpêtrièreParisFrance; 4 School of Biomedical Sciences, Li Ka Shing Faculty of MedicineUniversity of Hong KongSAR Hong KongChina; 5 Meta-Research Innovation Center at StanfordStanford UniversityStanfordUnited States; 6 Department of ImmunologyErasmus University Medical CenterRotterdamNetherlands; 7 Biological Computation & Process Lab, Chemical Process & Energy Resources InstituteCentre for Research & Technology HellasThessalonicaGreece; 8 Bioinformatics Research Laboratory, Department of Biological SciencesUniversity of CyprusNicosiaCyprus

**Keywords:** scientific language, etymology, history of science, None

## Abstract

The linguistic foundations of science and technology include many terms that have been borrowed from ancient languages. In the case of terms with origins in the Greek language, the modern meaning can often differ significantly from the original one. Here we use the PubMed database to demonstrate the prevalence of words of Greek origin in the language of modern science, and call for scientists to exercise care when coining new terms.

## Our etymological legacy

Plato once wrote that *"*the knowledge of names is a great part of knowledge*"* (Silverman, 1992). However, it is seldom that scientists or engineers think about the deeper origins of many of the names and words they use in their professional lives. These specialized vocabularies are introduced during high school and university to describe concepts, phenomena, methodologies and techniques, and we grow so accustomed to them that we lose sight of where they came from (Banay, 1948; Askitopoulou et al., 2000; Ramoutsaki et al., 2002; Danchin, 2010). For example, the word machine derives from the Greek word 'μηχανή', meaning 'trick', which is quite different from its modern usage. The study of the origins of words is called etymology, which, by the way, is derived from the Greek word 'έτυμος' meaning 'real' and 'genuine'.

It is widely accepted that the Greek language has provided more of these names and words than any other language (Flood, 1960; Silverman, 1992; Russo, 2004). As scientists and native speakers, we humbly urge our colleagues to delve a little deeper into the etymology of scientific terms of Greek origin and examine their meaning (Tamis, 2016). Non-Greek speakers will, we are sure, be surprised by the richness and structure of the Greek language, despite its often inept naturalization in English or other languages, and as a result be better able to understand their own areas of science (Snell, 1960; Montgomery, 2004). Our favorite example is the word 'analysis': everyone uses it, but few fully understand it. 'Lysis' means 'breaking up', while 'ana-' means 'from bottom to top' but also 'again/repetitively': the subtle yet ingenious latter meaning of the term implies that if you break up something once, you might not know how it works; however, if you break up something twice, you must have reconstructed it, so you must understand the inner workings of the system.

Many scientific words end with the suffix -ics, such as mathematics, physics and economics. However, these words were initially adjectives, not nouns, so strictly speaking a word like mathematics is incomplete in ancient Greek: the correct term is mathematical philosophy (words that are derived from Greek words meaning 'learnable' and 'love for wisdom' respectively; Nidditch, 1983). We are not arguing here that we need to use any scientific words and terms differently: rather, we are asking readers to be aware of the origins of the words they use and to think about these issues when proposing new scientific terms (Steffanides, 1965; Jarvis, 1996; Lewis, 2004; Welch, 2009).

## Word counts and a way forward

The PubMed database can be used to get an idea of the prevalence of words of Greek origin in the scientific literature: as of July 28, 2018 PubMed contained records for over 28 million scientific articles. We will focus here on frequently used terms (Newman et al., 2009), but that does not mean that the origins and use of other terms are not important. There are 243 distinct words that each appear in more than one million PubMed records (Supplementary file 1). However, when we exclude articles (such as 'the'), prepositions (such as 'to' and 'in') and short verb forms (such as 'is' and 'was'), we are left with 172 terms (Supplementary file 2), and removing terms with fewer that four characters (such as DNA and other abbreviations), leaves us with 152 terms (Supplementary file 3; Supplementary file 4). It is worth noting that no single gene or protein name appears in more than one million records (Seringhaus et al., 2008). When combined into a disjoint query, using the OR operator, these 152 terms retrieve ~27.8M entries, the majority (~97%) of all PubMed records.

From this initial dataset, we used etymological dictionaries to identify 15 terms with Greek origin: analysis, based, clinical, diagnosis, gene, genes, method, period, plasma, protein, proteins, surgery, system, therapy, type. The alert reader will notice that 13 of these words are nouns (with the two exceptions being the words 'clinical' and 'based'). We then constructed a PubMed search for records that contained at least one of the 15 words and it returned more than 23 million records, which is more than 80% of the entire database! We also constructed a PubMed search for records that contained at least one of the 137 non-Greek million-plus words and excluded all 15 words of Greek origin and it returned just 4.7 million records, confirming the prevalence of words of Greek origin in the scientific literature. It should be noted that these searches might underestimate the prevalence of Greek terms because, for example, some of the 137 words (such as mice) might be remotely connected by etymology to Greek words.

Advances in science mean that there is an ongoing need for new words (also known as neologisms). However, when a scientist attempts to introduce a new name for a new concept or idea, we would like him or her to consider both the origins of the new name as well as how it relates to existing names (Jackson, 1961; Trüper, 1999). In the field of genomics, the word epigenomics is an example of a new term being introduced in a thoughtful and meaningful way. However, there are many examples of researchers introducing new variations on the word genomics that are sub-optimal: Jonathan Eisen of the University of California, Davis has coined the term #badomics to describe such words.

A case in point is the term metagenomics, which is the study of genetic material taken from environmental samples. Here the prefix 'meta-' is used in a way that is not consistent with its Greek origins (meta- meaning beyond). Better names might have been endogenomics for the study of environmental samples taken from inside a host organism, and exogenomics for the study of samples taken from the outside (endo- and exo- meaning 'inside' and 'outside' in Greek, respectively). A similar situation might have occurred when looking for a name to describe the search for life elsewhere in the universe: the term exobiology was gradually replaced by astrobiology (Gargaud et al., 2011) – possibly because it had to rhyme with astronauts!

The suffix -some (from 'soma', meaning 'body' in Greek) has been employed in neologisms across the life sciences in recent decades, with varying degrees of success (Table 1). Likewise, the suffix -ome (from '-oma', meaning an undefined set in Greek) has been much used (think biome and genome) in recent times. To exemplify the linguistic richness of the Greek language, we explored the use of Greek prepositions to convey additional meaning within genomics. Epigenome (Murrell et al., 2005) is an excellent example of a scientific term in which a Greek preposition ('epi'-, meaning 'on top of') is combined with the suffix -genome to make a word that is precise and logically consistent. Other (less well known) examples include antigenome (which is used in immunology; Sette et al., 2016), metagenome (Streit and Schmitz, 2004), progenome (Ferreira et al., 2004), and hypergenome (Sgaramella, 2013). We then went on to create new words of this kind (Table 2) that, we feel, remain true to their etymological roots while, at the same time, being potentially useful to the scientific community. (The term pangenome (Tettelin et al., 2005), meaning the full complement of genes in a clade, is also very useful although, strictly speaking, 'pan-' is not a preposition).

**Table 1.** A selection of terms ending with the suffix -some that appear in the scientific literature. The term prostasome, which first showed up in PubMed in 1982, appeared in 218 PubMed records as of July 28, 2018. However, other terms ending with -some have proved much less popular.

Term	Definition	Context	Number of PubMed records	Year of first appearance
catansome	catanionic vesicle	synthetic biochemistry, surfactants	2	2008
ejectisome	extrusive organelle	cell biology and physiology	16	1984
histrosome	a type of ejectisome	cell biology and physiology	1	2015
hyposome	cellular structure	dDinoflagellate biology	7	2010
prostasome	prostate gland vesicle	sperm mobility and physiology	218	1982
remosome	remodeled nucleosome	non-canonical chromatin structure	2	2010

**Table 2.** A selection of terms that combine a Greek preposition and the suffix -genome. Some terms that the authors believe could be useful in genome biology.

Preposition	Term	Possible definition/interpretation
ana	anagenome	could be used to describe usage over time, to monitor population variation
amphi	amphigenome	could be used to describe polyploid genomes, and sex differences
apo	apogenome	could be used to convey non-DNA large scale analysis
dia	diagenome	could be a useful concept for comparative genomics
eis	eisgenome	could be a useful term for substance use
ek	ecgenome	alternative for exo-genomics (see text)
en	engenome	alternative for endo-genomics (see text)
kata	katagenome	could be a vey useful term to describe developmental processes over time
para	paragenome	could be used to describe the genomics of paralogs (although this is not satisfactory from an etymological point of view)
peri	perigenome	could be a very useful term to describe developmental processes over space
pros	prosgenome	could be used to describe synthetic genomes
syn	syngenome	could be used to describe the genomics of symbioses
hypo	hypogenome	could be used to describe a synthetic genome with depleted functions – *as opposed to hyper-genome to describe a synthetic genome with added functions*

## Conclusion and epilogue

It is often forgotten that some of humankind's greatest achievements in science, engineering, literature, philosophy, arts and architecture were communicated in Greek, not only in the ancient world but also more recently: the Greek language greatly contributed to the development of the Renaissance, to the French and American Revolutions, and to modern science. Scholars who spoke and wrote in Greek include Newton, Leibniz, Goethe and Wittgenstein, and Greek lives on (alongside Latin) in the taxonomic names devised by Linnaeus, Darwin and others. Perhaps we can still learn something from them, and from scholars in other fields, empowered by the availability of automated translation, online dictionaries and etymology tools. We hope that this contribution will encourage scientists to think about the terminology used in modern science, technology and medicine (Wulff, 2004), and to be more careful when seeking to introduce new words and phrases into our vocabulary.

## Data Availability

The supplementary files for this paper and other related information is available at: https://doi.org/10.6084/m9.figshare.5493133.v2. Etymology of the select terms of Greek origin: http://troodos.biol.ucy.ac.cy/Etymology.html.

## Additional files

Supplementary file 1. The 243 words that appear in at least one million PubMed records.

Supplementary file 2. The 172 words with rich meaning (nouns, adjectives and verb forms) that appear in at least one million PubMed records. A part-of-speech tagger was used to classify the words under consideration (https://cst.dk/tools/index.php).

Supplementary file 3. The 152 words with rich meaning and four or more characters that appear in at least one million PubMed records.

Supplementary file 4. A search string containing the 152 words with rich meaning and four or more characters that appear in at least one million PubMed records. The search that excludes the 15 Greek terms is generated automatically at the following URL: https://tinyurl.com/y7kflbcb
